# Insights Into the Known ^13^C Depletion of Methane—Contribution of the Kinetic Isotope Effects on the Serine Hydroxymethyltransferase Reaction

**DOI:** 10.3389/fchem.2021.698067

**Published:** 2022-01-05

**Authors:** Gerd Gleixner

**Affiliations:** Max-Planck-Institut für Biogeochemie, Jena, Germany

**Keywords:** intramolecular isotope distribution, carbon isotope (*δ* ^13^C), kinetic isotope effect (KIE), serine, methane

## Abstract

We determined the kinetic isotope effect on the serine hydroxymethyltransferase reaction (SHMT), which provides important C_1_ metabolites that are essential for the biosynthesis of DNA bases, O-methyl groups of lignin and methane. An isotope effect on the SHMT reaction was suggested being responsible for the well-known isotopic depletion of methane. Using the cytosolic SHMT from pig liver, we measured the natural carbon isotope ratios of both atoms involved in the bond splitting by chemical degradation of the remaining serine before and after partial turnover. The kinetic isotope effect ^13^(V_Max_/K_m_) was 0.994 0.006 and 0.995 0.007 on position C-3 and C-2, respectively. The results indicated that the SHMT reaction does not contribute to the ^13^C depletion observed for methyl groups in natural products and methane. However, from the isotopic pattern of caffeine, isotope effects on the methionine synthetase reaction and on reactions forming Grignard compounds, the involved formation and fission of metal organic bonds are likely responsible for the observed general depletion of “activated” methyl groups. As metal organic bond formations in methyl transferases are also rate limiting in the formation of methane, they may likely be the origin of the known ^13^C depletion in methane.

## Introduction

The stable isotopes of the bioelements (H, C, N, O) are not statistically distributed in natural compounds, but represent specific intramolecular isotope distributions (Schmidt et al., 1995). Many reasons for these distributions are suggested. In 1985, the geochemist E. Galimov proposed thermodynamic reasons. Presuming a total energy equilibration between all atoms of a given system of molecules, which is likely in the “slow” and “energy-rich” rock cycle, Galimov suggested that isotope effects on chemical bond enthalpies explain these isotopic patterns (Galimov, 1985). His calculated “*β*-factors” were in line with the isotopic patterns of some compounds known at that time (Abelson and Hoering, 1961; Meinschein et al., 1974; Rinaldi et al., 1974); however, they were not in agreement with patterns observed in natural products (Schmidt et al., 1995). Obviously, the presumption that all molecules and all atoms within these molecules are in thermodynamic binding equilibrium is not valid for biological systems. Here, kinetic isotope effects on enzymatic reactions are expected to contribute to isotopic discrimination. This has widely been demonstrated for the primary CO_2_ fixation reactions (O'Leary, 1981; Winkler et al., 1982; Farquhar, 1983; WinklerSchmidt et al., 1983), and as an example in secondary metabolism, it has been shown that the isotope effect on the pyruvate dehydrogenase reaction is responsible for the general depletion of ^13^C in metabolites of acetyl-CoA, such as fatty acids or isoprenoids (Park and Epstein, 1960; Whelan et al., 1970; DeNiro and Epstein, 1977; Winkler and Schmidt, 1980; Monson and Hayes, 1982; Melzer and Schmidt, 1987; Schmidt et al., 1995). In addition, it has been demonstrated that the formation of ^13^C depleted lipids has to be compensated by ^13^C enrichments in other compounds (Abelson and Hoering, 1961; Epstein, 1968). Furthermore, investigations on the isotopic pattern of glucose and its metabolites (Roßmann et al., 1991; Gleixner et al., 1993) demonstrate the importance of the isotopic pattern of the metabolic precursor and the influence of turnover rates at metabolic branching points (Schmidt et al., 1995) for the observed pattern of a natural product.

The reason for well-known ^13^C depletions in biological *N*- and *O*-methyl groups of lignin (Galimov et al., 1976; Keppler et al., 2004) and other biological compounds like methane (Krueger and Krueger, 1984; Caer et al., 1991; Schmidt et al., 1995; Weilacher et al., 1996), however, involves several possible reactions. A common starting ground is that all depleted methyl groups originate from the C_1_ metabolism. This pool is fed by the serine hydroxymethyltransferase (SHMT) reaction, and it was suggested that an isotope effect on this reaction could be responsible for the ^13^C depletion found for CH_3_-groups in natural products.

**FIGURE 1:**
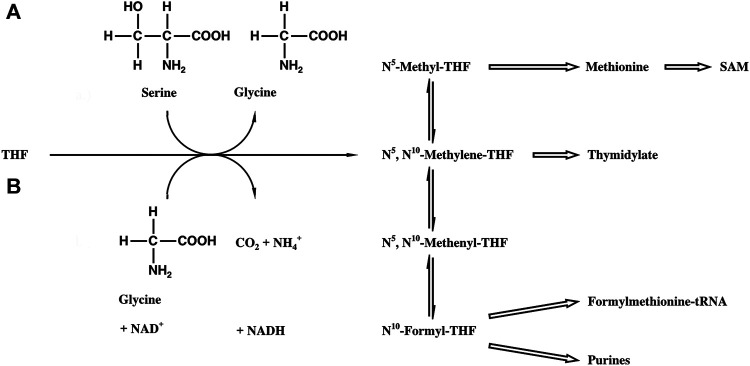
Biosynthetic scheme of the THF-bound C_1_ pool. **(A)** SHMT reaction. **(B)** Glycine synthase reaction.

SHMT [EC 2.1.2.1] catalyzes the reversible cleavage of serine to glycine and tetrahydrofolic acid (THF) bound formaldehyde (N^5^, N^10^-Methylene-THF, Figure 1). Methylene THF is the most important source for the C_1_ metabolism, which is essential for cell proliferation (Rao et al., 1987; Stover and Schirch, 1993). The pool provides C_1_ units for the biosynthesis of purines, thymine, formylmethionine tRNA, and methionine, the latter being the precursor of *S*-adenosylmethionine (SAM), the biological agent for methylations. Correspondingly, SHMT is one of the most ubiquitous enzymes in nature (Schirch, 1982), being found in all organisms (fungi, bacteria, insects, and plants), in different organs of animals (liver, kidney, and brain), and in different cell compartments (cytosol and mitochondria).

In our experiments, we used the cytosolic SHMT from pig liver, which is a homotetramer with a molar mass of 212 kD. Each subunit has covalently bound pyridoxal 5′-phosphate (PLP), responsible for the yellow color of the enzyme. The enzyme catalyzes in its “closed” form, which is supposed to be the physiological one, the transfer of C1 units to THF, but it also catalyzes in its “open” and probably not physiological form THF independent reactions like aldol cleavages, transaminations, and decarboxylations (Schirch, 1982). For the reaction catalyzed by the “closed” form, three different mechanisms are discussed including a retroaldol cleavage, the formation of a thiohemiacetal, or the direct nucleophilic attack of THF (Matthews and Drummond, 1990). The study of the carbon isotope effect on the SHMT reaction could contribute to the distinction between these discussed enzyme mechanisms, even though from our point of view the role of the enzyme in the depletion of methyl groups would be of primary importance.

## Materials and Methods

### Chemicals

All ordinary chemicals were of analytical grade and purchased from local suppliers. 5,6 (*R*,*S*),7,8-THF C_19_H_23_N_7_O_6_*2HCl*2H_2_O was purchased from Boehringer Mannheim GmbH (Mannheim, GER) and *l* -Serine from Fluka AG (Buchs, CH). 6(*S*)-THF was produced from racemic folic acid by chemical reduction to 7,8 dihydrofolic acid and by reduction with dihydrofolic acid reductase to 5,6(*S*),7,8-THF and was donated from V. Schirch, Medical College of Virginia, United States (Stover and Schirch, 1992).

### Enzymes

The cytosolic SHMT [EC 2.1.2.1] from pig liver with an activity of 0.43 μkat/mg, dihydrofolate reductase [EC 1.5.1.3], and “trifunctional enzyme” TFE (methylenetetrahydrofolate dehydrogenase [EC 1.5.1.5], methenyltetrahydrofolate cyclohydrolase [EC 3.5.4.9], and formyltetrahydrofolate synthetase [EC 6.3.4.3]) with 0.03 μkat/mg dehydrogenase activity were isolated, and enzyme activity was determined as described previously (Stover and Schirch, 1992). Methylenetetrahydrofolate reductase (MTHFR) was isolated as described previously from pig liver with an activity of 0.14 μkat/ml (NADPH-CH_2_-H_4_folate oxidoreductase assay in water, Matthews, 1986). All enzymes were donated by V. Schirch, Medical College of Virginia, United States.

### Determination of the Kinetic Isotope Effect on the SHMT Reaction

**FIGURE 2:**
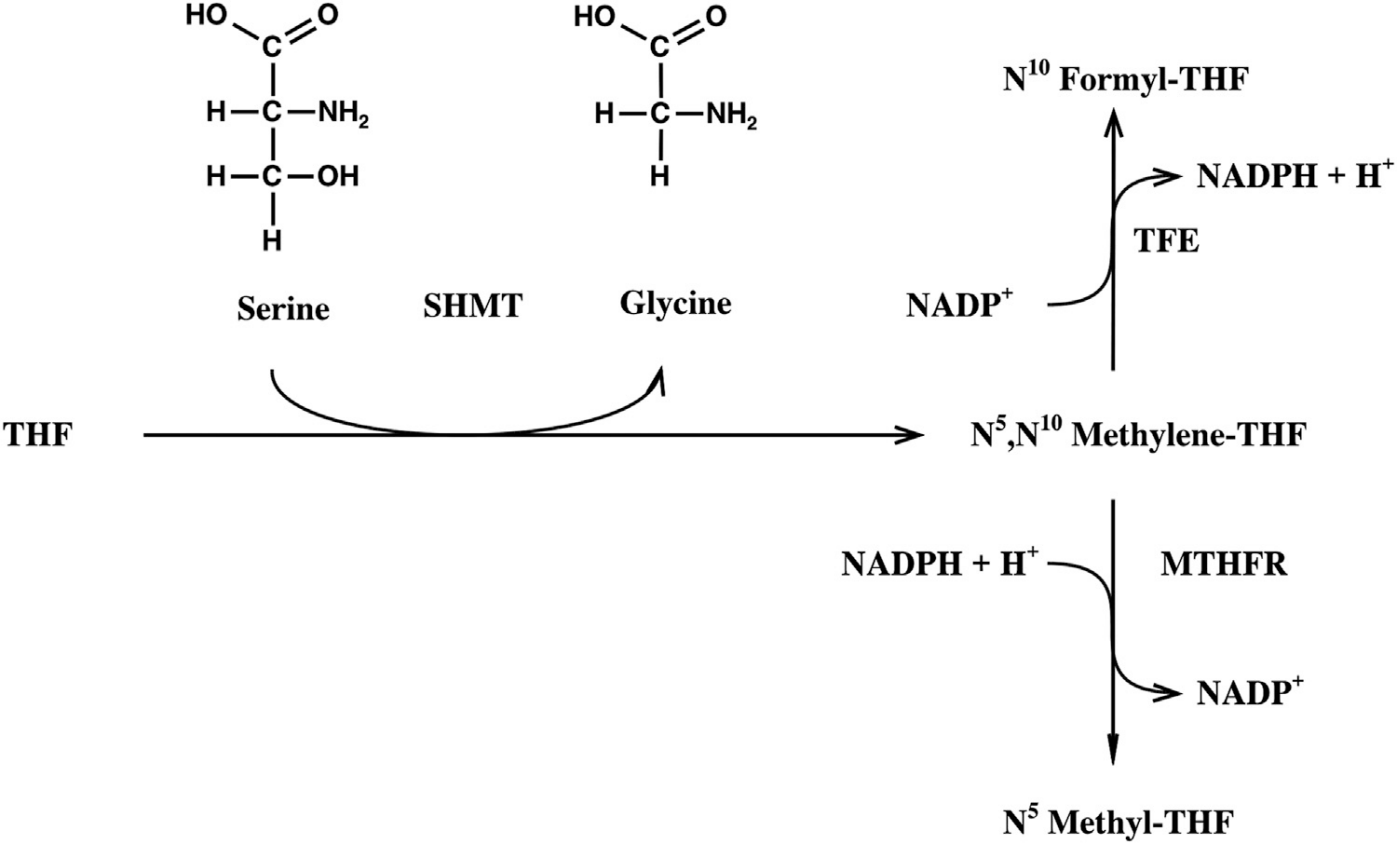
Alternative reaction assays for the determination of the kinetic isotope effect on the SHMT reaction. THF, tetrahydofolic acid; SHMT, serine hydroxymethyltransferase; TFE, trifunctional enzyme; MTHR, methylene THF reductase.

**FIGURE 3:**
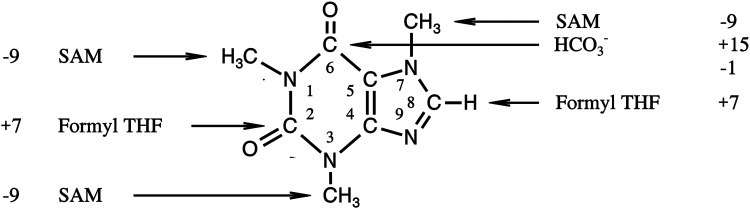
Origin of different positions in caffeine and their difference in [‰ (mUr)]_PDB_ to the mean δ^13^C-value of whole the molecule (see 22).

The competitive method was used throughout (O´Leary, 1980). Here, the enzymatic reaction that is investigated is coupled to a second enzymatic reaction that removes the formed product immediately in order to make the first reaction irreversible. The remaining substrate after partial enzymatic turnover (50%) is analyzed. The irreversibility of the SHMT reaction was guaranteed by enzymatic oxidation or reduction of the methylene THF formed to formyl THF or methyl THF, respectively (Figures 2, 3). The turnover of the reaction was determined on the basis of the absorbance of NADPH at 340 nm. In the oxidative assay 1,000 µl 20 mM KH_2_PO_4_ buffer (pH 7.3) containing 45 mM *β*-mercaptoethanol, 50 µl 10 mM NADP^+^ solution, 50 µl 24.4 mM 6*S*-THF solution, 0.13 µkat TFE, and 0.25 nkat SHMT were incubated at 25°C. An aliquot of 300 µl was used in a 1 mm quartz cuvette to measure the baseline at 340 nm (Varian UV/VIS spectrometer). The assay was started in the joint solutions by adding 1 µmol serine. The reductive assay was performed analogously, but 50 µl 12.5 mM NADPH solution and 14 nkat of MTHFR were used.

An aliquot of the reaction mixture (300 µl) was again transferred to a quartz cuvette (1 mm) in order to control the turnover rate on the basis of NADPH absorption (*ε*_340_ = 6230 M^−1^ cm^−1^). After a turnover of 50–70%, the remaining solution was heated to 100°C to denature the enzymes, and the solution was concentrated to a volume of 500 µl.

The enzymatic experiments were performed at the Medical College of Virginia, United States under the supervision of V. Schirch and R.G. Matthews.

### Isolation of Serine

The residual serine was isolated from the assay medium by HPLC (Perkin Elmer) in the Medical College of Virginia. The solution (500 µl) was applied to a LiChroSorb CX 10 µm cation exchange column (outer diameter 4.6 x column length 250 mm, Merck (Darmstadt, GER)). During the isocratic (H_3_PO_4_ pH 2.8) separation, serine was monitored through its absorption at 210 nm and finally identified from the collected eluate (1 ml) by its UV spectrum (HP 1050, photodiode array spectrometer). Serine containing fractions (retention time ca. 20 min) were pooled (3–5 ml) and stored at −70°C.

### Degradation of Serine, Positional Isotope Analysis, and Calculation of Isotope Effects

For the chemical degradation of isolated serine and the isotopic analysis of its fragments, 2 µl 500 mM KH_2_PO_4_ buffer pH 5.8, containing 100 nmol serine, 300 nmol NaIO_4_ in 2 µl H_2_O were added in a closed autosampler vial (700 µl); the mixture was incubated for 15 min at 65°C. Under these conditions, periodate degrades serine into CO_2_ (C-1), formic acid (C-2), and formaldehyde (C-3) (Simon and Floss, 1967; Gleixner, 1994). One microliter of this reaction mixture was transferred to the GC-C-IRMS system (Isochrom 1, VG Isogas (Middelwich, GB)). The reaction products were separated on a PoraPLOT U capillary column (Chrompack (Frankfurt, GER), outer diameter 0.32 mm, column length 5 m) using a temperature program (2 min 110, 0.3°C/min to 140°C) and oxidized by online combustion (CuO, 800°C). The CO_2_ formed was directly transferred to the IRMS and measured against bottled CO_2_ monitor gas that was calibrated against NBS 19. All isotope ratios are expressed as ^13^C-values (Coplen, 2011).

The kinetic isotope effect on the SHMT reaction ^13^(V_Max_/K_m_) was determined from the isotope ratio of serine in position C-3 before and after partial turnover (R_0_ and R_S_) and the turnover fraction f were used:

$${}^{13}\left(V_{\max}/K_{m}\right)=\log(1-f)/\log\left[\left(1-f\right)*\left(R_{s}/R_{0}\right)\right]$$

## Results

### Principle of the Determination of the Kinetic Isotope Effect on the SHMT Reaction

The assay developed was based on the enzymatic assay of Vanoni et al. (Vanoni et al., 1990) for the determination of the secondary tritium isotope effect on the SHMT reaction. Serine was split by SHMT into glycine and methylene THF. The latter was either immediately reduced by methylene THF reductase and NADPH to methyl THF, or oxidized by TFE and NADP^+^ to formyl THF (Figure 2). The activity of the auxiliary enzymes was always far higher than that of the SHMT, in order to guarantee that this reaction was rate limiting and the whole reaction sequence irreversible. Results obtained with both alternatives were identical; however, as handling of TFE and NADP^+^ was more convenient, most assays were performed with the SHMT and TFE method. The isotope effect was determined on the basis of the isotopic pattern analysis of the substrate at 0% and of the remaining substrate after partial turnover (50–70%); for this purpose, the residual serine was isolated from the medium by cation exchange. The amino acid was submitted to NaIO_4_ fission, and the isotope ratios of the fission products were determined by GC-C-IRMS.

### Check for Possible Isotope Effects on the Method Itself

The kinetics, yield, and reproducibility of the serine degradation by periodate were controlled on mmolar samples of pure serine measuring the amount of CO_2_ formed. This product was cryogenically purified and determined gas volumetrically. Between 92 and 99% yield were found. Independent of the sample size (nmol - mmol), an identical isotope ratio of the CO_2_ was found (0.2–0.5‰ (mUr) difference).

Furthermore, the degradation method was checked for isotope effects from an isotope balance, comparing the total isotope ratios before and after degradation. After serine degradation in nmol amounts, the mean isotope ratio of the fragments was identical to the measured isotope ratio of the whole molecule (Table 1). Finally, in order to check the isolation method for isotope effects, serine was degraded before and after chromatographic purification from incubation medium, and the isotope ratios of the fragments were compared (Table 1). Differences of 2 and 7‰ (mUr) were found for positions C-1 and C-2, respectively, but no difference for position C-3, the only relevant one in regard to the one carbon pool. Therefore, the method developed is suitable for the determination of the kinetic isotope effect on the SHMT reaction.

**TABLE 1:** *δ* ^13^C-values of serine and of its molecule positions before and after isolation from incubation medium.

Serine	δ^13^C-value [‰ (mUr)]_PDB_ in position				
	Bulk measured	Bulk calculated	C-1	C-2	C-3
Incubated	−11.7 ± 0.1	−11.9	−15.3 ± 1.7	−5.0 ± 0.6	−15.4 ± 1.4
Isolated	—	−14.7	−17.5 ± 1.9	−	−

### Results and Calculation of the Kinetic Isotope Effect on the SHMT Reaction

The mean calculated kinetic isotope effect (KIE) on position C-3 of serine was ^13^(V_Max_/K_m_) = 0.994 0.006 (Table 2), which is in the range of a secondary isotope effect.

**TABLE 2:** Isotope ratio of position C-3 in serine before and after incubation with SHMT and calculated kinetic isotope effect on the reaction.

Turnover	Isotope ratio in position C-3 of serine		Kinetic isotope effect
f	Isolated	Incubated	
0.2	0.011038	0.011074	0.9856
0.5	0.011012	0.011074	0.9920
0.51	0.011055	0.011074	0.9976
0.54	0.010994	0.011074	0.9908
0.63	0.011106	0.011074	1.0029
0.80	0.011002	0.011074	0.9960
—	—	mean	0.9941 ± 0.0060

For the calculation of the KIE on positions C-1 and C-2, the measured δ^13^C-value shifts of these positions after the reaction were corrected for the shifts observed during the separation step (Table 3). The resulting KIE on position C-1 and C-2 of serine were ^13^(V_Max_/K_m_) = 0.995 0.005 and 0.995 0.007, respectively. Hence the SHMT reaction does not appear to contribute to the observed carbon isotope discrimination.

**TABLE 3:** Isotope ratio of position C-1 and C-2 in serine before and after incubation with SHMT and calculated kinetic isotope effect on the reaction. Isotope ratios of the isolated products were linear corrected for the isotope effect of the separation step.

Turnover	Isotope ratio in position			
f	Isolated		Incubated		Kinetic isotope effect	
	C-1	C-2	C-1	C-2	C-1	C-2
0.2	0.011050	0.011092	0.011041	0.011102	1.004	0.996
0.5	0.010993	0.010993	0.011041	0.011102	0.994	0.986
0.51	0.010960	0.011020	0.011041	0.011102	0.990	0.990
0.54	0.010964	0.011034	0.011041	0.011102	0.991	0.992
0.63	0.011011	0.011155	0.011041	0.011102	0.997	1.005
0.80	0.010979	0.011108	0.011041	0.011102	0.997	1.000
—	—	—	—	—	0.995	0.995
—	—	—	—	—	±0.005	±0.007

## Discussion

The measured isotope effects on the SHMT reaction have to be discussed in order to identify the rate determining step of the SHMT reaction, and to interpret the strong ^13^C depletions known for certain C_1_ metabolites (Schmidt et al., 1995).

Concerning the first aspect, it has to be pointed out that the isotope effect found on the two carbon atoms involved in the bond cleavage catalyzed by the SHMT reaction, as well as that on the C-1 (not involved in the bond cleavage) are identical and close to unity (KIE for C-1 = 0.994, C-2 = 0.995, C-3 = 0.995). This result is in line with findings of Vanoni et al. (Vanoni et al., 1990) who did not observe any secondary tritium isotope effect on the reaction. Both results therefore suggest that the C-C bond splitting is not rate limiting for the reaction, and that substrate dissociation is slower than the chemical steps of the reaction sequence, resulting in a high forward commitment (c_f_) to catalysis. Given the “closed” conformation of the SHMT, as proposed for this reaction, a high c_f_ is reasonable. Therefore, the results cannot contribute to a further distinction of the alternative chemical mechanisms discussed for the enzyme reaction.

More informative and important as to our main point of view is the fact that the SHMT reaction obviously does not contribute to the observed ^13^C depletions of methyl groups in natural products. This finding has therefore to be discussed taking additionally into account the observed isotope pattern of caffeine, which we described elsewhere (Weilacher et al., 1996). In the core of this heteroaromatic purine alkaloid (Figure 3), two carbon atoms derive from the THF-bound C_1_ pool and three others, the methyl groups, from the SAM-bound C_1_ pool. The latter C atoms were depleted of ^13^C relative to those from the THF-bound C_1_ pool by 16‰ (mUr) (Figure 3). This discrimination, combined with our results on the isotope effect on the SHMT reaction, indicate unequivocally an isotope effect in the transfer of C_1_ units from the THF-bound C_1_ to the SAM-bound C_1_ pool. A kinetic isotope effect ≥1.017 for a corresponding reaction can be estimated from the δ^13^C-values of the representatives in the two C_1_ pools.

**FIGURE 4:**
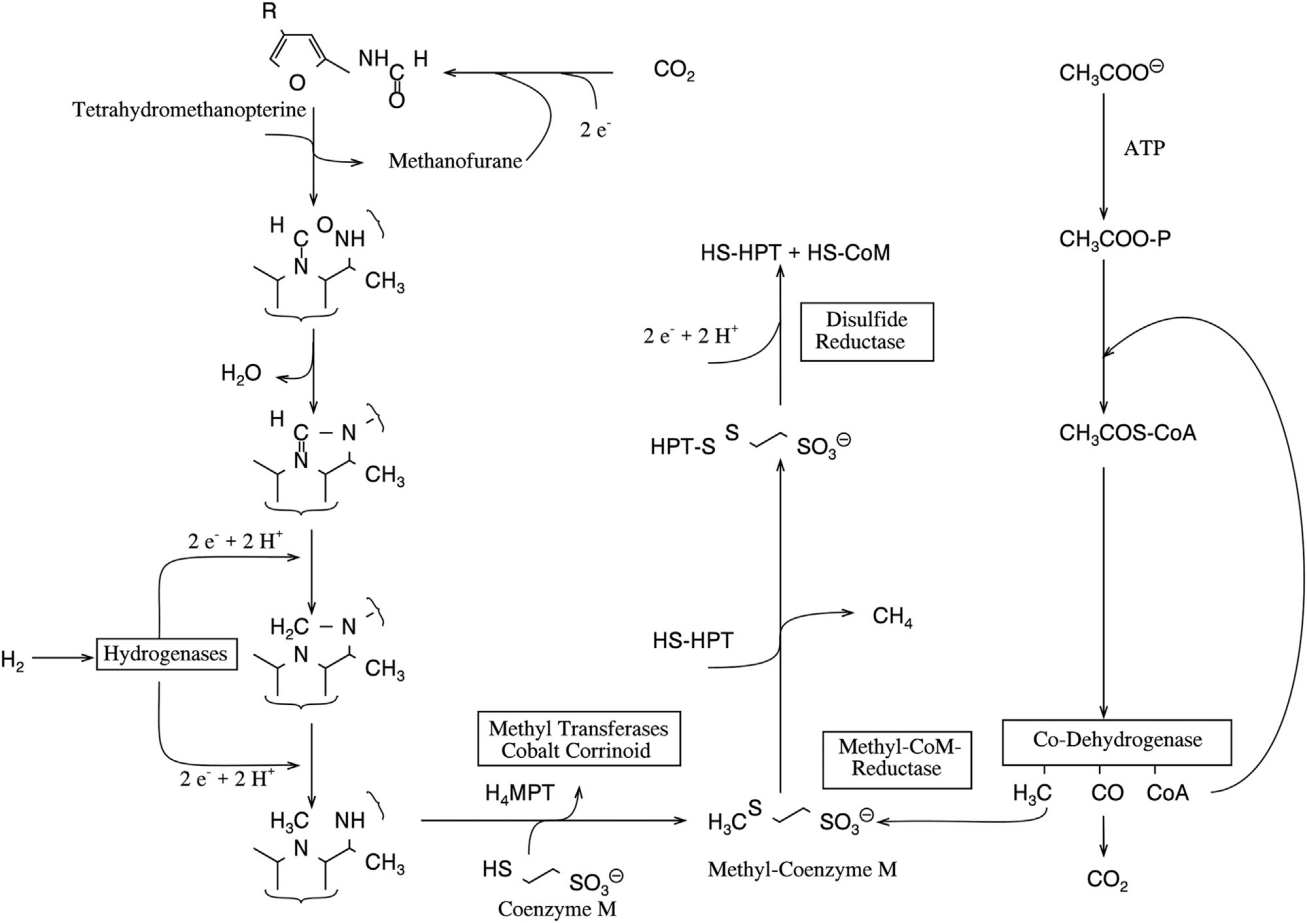
Biological methanogenesis starting from CO_2_ and acetate (adapted after Jaun, 1993).

This conclusion must have a general importance for isotope fractions in C_1_ metabolism. Methane liberated in wetlands and swamps can be depleted up to -110‰ (mUr) (Hoefs, 1987). Quite remarkable ^13^C depletions are also reported for methane produced by bacteria (Fuchs et al., 1979). For bacterial methanogenesis, two sources are known, namely, CO_2_ and acetate (Thauer et al., 1985; Wolfe et al., 1990, see Figure 4). The pathway of methane biosynthesis from CO_2_ has in certain phases a strong analogy to the reduction of C_1_ units for the THF pool; however, for energetic reasons, the CO_2_ is bound to tetrahydromethanopterin, a cofactor with a lower redox potential than THF.

CO_2_ was also the only source for methane in previous investigations of our group on simulated rumen fermentation (Metges et al., 1990). From the molar balance of the substrate and the fermentation products, it was possible to calculate the amount and the δ^13^C-value of the primarily produced CO_2_, which was partially reduced for the formation of methane. Its turnover and the δ^13^C-values of the remaining CO_2_ and the formed methane (−9‰ (mUr) and −70‰ (mUr), respectively) yielded a discrimination corresponding to an “isotope effect” of 1.06 for the bacterial methanogenesis. This value is much higher than the isotope effect estimated for the transfer of C_1_ units from the THF to the SAM pool, but still below the isotope effect reported for the catechole-*O*-methyltransferase reaction (1.09) and the methionine synthase reaction (1.087) (Gray et al., 1979; Hegazi et al., 1979; Romek et al., 2017).

Nevertheless, all these findings indicate that isotope discriminations accompanying the formation or the transfer of methyl groups and not the formation of THF-bound C_1_ units cause the depletion. This has recently been shown for methionine synthase but is still unknown for the enzymes catalyzing the methyl transfer to coenzyme M for methanogenesis (Figure 4). All these enzymes have a cobalamin as prosthetic group and bind methyl units to this core metal *via* metal organic bonds. The proposal, that the observed ^13^C discrimination takes place in this bond fission, is supported by the finding that the release of hydrocarbons from Grignard compounds is also accompanied by a large carbon isotope effect (Holm, 1993; Vogler and Hayes, 1978). In consequence, we suggest that further investigations on the origin of the ^13^C depletion in methane focus on corresponding reactions.

## Data Availability

The original contributions presented in the study are included in the article/Supplementary Material. Further inquiries can be directed to the corresponding author.

## Author's Note

This manuscript is dedicated to the 90th birthday of H.-L. Schmidt.
